# Liraglutide Regulates the Kidney and Liver in Diabetic Nephropathy Rats through the miR-34a/SIRT1 Pathway

**DOI:** 10.1155/2021/8873956

**Published:** 2021-04-05

**Authors:** Shan Xiao, Ye Yang, Yue-Tong Liu, Jun Zhu

**Affiliations:** ^1^Department of Endocrinology, People's Hospital of Shenzhen Baoan District, The Second School of Clinical Medicine, Southern Medical University, The Second Affiliated Hospital of Shenzhen University, Shenzhen, Guangdong, China; ^2^Department of No. 1 Cadres, The Second Affiliated Hospital of Xinjiang Medical University, Urumqi, Xinjiang, China; ^3^Department of Ultrasonic ECG, Sun Yat-sen University Cancer Center, Guangzhou, Guangdong, China

## Abstract

**Purpose:**

To explore the regulatory effects of liraglutide on the kidney and liver through the miR-34a/SIRT1 pathway with related factors in diabetic nephropathy (DN) rats.

**Methods:**

DN rats were randomly divided into two groups (*n* = 10) and were injected with liraglutide or normal saline twice a day. The 24-hour urine microalbumin content and biochemical index levels were measured. qRT-PCR was performed to detect the expression of miR-34a in the kidney and liver tissues. The levels of SIRT1, HIF-1a, Egr-1, and TGF-*β*1 in kidney and liver tissues were determined using qRT-PCR, western blot, and immunohistochemistry. Electron microscopy and HE staining were used to observe the ultrastructure and pathological changes.

**Results:**

Liraglutide treatment in DN rats decreased blood glucose, 24-hour urine microalbumin, TC, TG, LDL-C, UA, Cr, UREA, ALT, and AST levels and increased the level of HDL-C (*P* < 0.05). Compared with the control group, the miR-34a levels were significantly decreased in kidney and liver tissues followed by liraglutide treatment (*P* < 0.05). The levels of SIRT1 in the liraglutide group are significantly higher than those in the control group with the kidney and liver tissues (*P* < 0.05). Conversely, the contents of HIF-1a, Egr-1, and TGF-*β*1 were significantly lower in the liraglutide group than in the control group (*P* < 0.05). Electron microscopy showed that the kidney of the liraglutide-treated group exhibited minor broadening of the mesangial areas, fewer deposits, and a well-organized foot process. HE staining revealed that the kidney of the liraglutide-treated rats had a more regular morphology of the glomerulus and Bowman sac cavity and lighter tubular edema. Additionally, the liraglutide-treated DN rats had a clear hepatic structure, a lower degree of steatosis, and mild inflammatory cell infiltration.

**Conclusion:**

Liraglutide, through its effect on the miR-34a/SIRT1 pathway, may have a protective role in the kidney and liver of DN rats.

## 1. Introduction

Diabetes mellitus (DM) has now become a global health problem. Obesity or overweight, which is caused by lifestyle changes, is an important risk factor for type 2 DM (T2DM) [1, 2]. Diabetic nephropathy (DN) is a common microvascular complication of DM and is an important cause of end-stage renal disease. The pathogenesis of DN may be associated with abnormal renal hemodynamics, oxidative stress, and formation of glycosylation end products [3–6]. The primary pathological changes associated with DN include glomerular sclerosis, renal tubular atrophy, and renal interstitial fibers [7]. In addition to kidney disease, patients with DN may have abnormal liver metabolism [8]. Liraglutide, a glucagon-like peptide-1 (GLP-1) analog, has been proven to regulate lipid metabolism and is able to decrease blood glucose levels and body weight. Moreover, liraglutide improves urinary albumin levels, reduces kidney fibrosis, and delays DN progression [9–13]. Liraglutide also enhances the antioxidant capacity of hepatocytes by activating the NRF2 (nuclear factor erythroid 2-related factor 2) signaling pathway and reduces hepatocyte apoptosis that is mediated by glycolipid toxicity in diabetic and obese rats [14]. Recently, microRNAs (miRNAs) have become a research hotspot. miRNAs are endogenous, noncoding RNAs composed of 21-25 nucleotides. They suppress gene transcription or protein translation by targeting the 3′-noncoding region (3′-UTR) of the target mRNA [15]. The importance of miRNAs in regulating critical signaling pathways and their consequent role in the pathogenesis of DN have been demonstrated in various studies [16–19]. In particular, miR-34a has been implicated in the development of fibrosis in various organs and early pathological changes in the kidney in DN [20]. In renal tubular epithelial cells, p53 activates the transforming growth factor-*β*1 (TGF-*β*1) and induces overexpression of miR-34a, which aggravates the degree of renal fibrosis following the unilateral ureteral obstruction (UUO) in mice [21]. Alterations in miR-34a expression levels have been shown to be closely related to liver fibrosis; activation of the miR-34a/SIRT1 pathway plays a critical role in fructose-induced liver fibrosis via the TGF-*β*1/Smads pathway in rats [22]. In glomerular mesangial cells treated with high glucose, overexpression of miR-34a inhibits the expression of SIRT1 but increases the expression of the hypoxia-inducible factor-1 *α* (HIF-1a) and early growth response-1 (Egr-1); this causes inflammation of the glomerular mesangial cells as well as an increase in the levels of profibrotic factors [23, 24]. Hypoxia has been shown to be an important inducing factor of inflammation and fibrosis in chronic liver disease; HIF-1a is a key transcription factor for liver fibrosis and is involved in the biliary ligation (BDL) and carbon tetrachloride- (CCL4-) induced liver fibrosis in rats [25–27]. HIF-1a was present in elevated levels in the liver of T2DM rats with fatty liver disease. Egr-1 is a proinflammatory nuclear transcription factor; Egr-1 knockout mice were shown to exhibit more severe liver fibrosis, which was induced by treatment with acetaminophen (APAP) [28]. Obesity and T2DM are often accompanied by insulinemia; long-term stimulation with high insulin induces the activation of Egr-1 in hepatocytes and reduces the regulatory effect of insulin. Conversely, inhibiting the expression of Egr-1 in the liver significantly reduces glucose production, thereby resulting in an improvement in glucose tolerance and insulin sensitivity [29].

Therefore, we established a rat model of DN using streptozotocin (STZ) and explored the effects of liraglutide on the expression of miR-34a and SIRT1 in the kidney and liver tissues. This study was aimed at investigating whether liraglutide has a role in protecting the kidney and liver of DN rats through the miR-34a/SIRT1 pathway; hence, this study could provide a theoretical basis for the use of liraglutide in the prevention and treatment of DN.

## 2. Procedures

### 2.1. Animals

SPF-grade male Sprague Dawley (SD) rats, weighing approximately 180-220 g, were provided by the Laboratory of Animal Center of Xinjiang Medical University. This study was approved by the Institutional Animal Care and Use Committee (IACUC) of Xinjiang Medical University and performed in accordance with the rules and regulations of the Care and Use of Laboratory Animals.

### 2.2. Reagents

STZ was purchased from Sigma-Aldrich (USA); the TRIzol reagent was purchased from Ambion (USA); the cDNA reverse transcription kit and qRT-PCR kit were purchased from Qiagen (Germany); primers for SIRT1, HIF-1a, Egr-1, and miR-34a were synthesized by Tianyihuiyuan Biotech (China); antibodies against TGF-*β*1, SIRT1, HIF-1a, and Egr-1 were purchased from Abcam (USA); liraglutide was purchased from Novo Nordisk (Denmark).

### 2.3. Instruments

The instruments used in our study included a portable blood glucose monitor and test paper (Accu-Chek, Germany), a PCR amplifier CFX96 (Bio-Rad, USA), a gel imager and an electrophoresis system (Bio-Rad, USA), and an automatic chemiluminescence analyzer (Tianneng Technology, China).

### 2.4. Model Establishment

Twenty SPF-grade male SD rats were fed with a high-fat and high-sugar diet for 8 weeks until their body weight increased to 400-450 g. The high-fat and high-sugar diet was resumed once again after the rats were injected intraperitoneally with 1 dose of STZ (30 mg/kg). After 3 days, the tail vein blood was collected; blood glucose levels equal to or greater than 16.7 mmol/L were used as an indicator for the successful establishment of a diabetic model. A DN model was considered to be successfully established if the urine albumin excretion rate exceeded 20 mg/24 h and the urine sample tested positive for glucose after 4 weeks. Thereafter, the DN rats were randomly divided into 2 groups (*n* = 10) and were subcutaneously injected with liraglutide (6 mg liraglutide dissolved in 11 mL normal saline, 0.6 mg/kg/d) or normal saline twice a day, then fed a high-fat and high-sugar diet. The rats belonging to both the liraglutide and control groups were euthanized by an anesthetic overdose after 12 weeks.

### 2.5. Sample Collection

Following 12 weeks of treatment with liraglutide and normal saline, 24-hour urine samples were collected. Following an overnight fast, the rats were weighed and intraperitoneally injected with 3% pentobarbital sodium (40 mg/kg). Blood was collected from the abdominal aorta; a portion of each of the kidney and liver tissues was kept in RNAlater solution and stored at -80°C. Another portion was fixed with 4% paraformaldehyde and 2.5% glutaraldehyde for use in subsequent experiments.

### 2.6. 24-Hour Urinary Microalbumin

The 24-hour urine samples were collected and centrifuged at 2500 × g for 20 min. The supernatants were collected, and the absorbance was measured at a wavelength of 450 nm using a microplate analyzer. The microalbumin content of the urine was calculated according to the regression equation.

### 2.7. Serum Biochemical Indices

The blood samples were thawed at 4°C, and GLU, total cholesterol (TC), triglyceride (TG), low-density lipoprotein (LDL-C), high-density lipoprotein (HDL-C), uric acid (UA), creatinine (Cr), urea (UREA), alanine aminotransferase (ALT), and aspartate aminotransferase (AST) contents in the blood samples were determined using a BS-420 automatic biochemical analyzer.

### 2.8. Quantitative Real-Time PCR (qRT-PCR)

Total RNAs from the kidney and liver tissues were extracted using the TRIzol reagent and reverse transcribed to cDNA. Primers for SIRT1, HIF-1a, Egr-1, and TGF-*β*1 are shown in Table 1. The PCR reaction included 40 cycles of denaturation at 95°C for 10 min, annealing at 60°C for 1 min, and extension at 95°C for 15 s. Glyceraldehyde 3-phosphate dehydrogenase (GAPDH) was used as the internal reference. The expression levels of miR-34a were determined by performing a PCR reaction consisting of 40 cycles of denaturation at 94°C for 15 min, annealing at 55°C for 30 s, and extension at 70°C for 30 s. The has-U6 was used as the internal reference. Each experiment was repeated at least 3 times. The relative levels of SIRT1, HIF-1a, Egr-1, TGF-*β*1, and miR-34a were determined using the 2^-*ΔΔ*Ct^ (cycle threshold (CT)) formula.

### 2.9. Western Blot

The kidney and liver tissue samples were sonicated in the lysis buffer containing a phosphatase inhibitor and protease and were subsequently centrifuged at 14000 × g for 15 min at 4°C, The supernatants were collected, and the protein concentration was determined using a bicinchoninic acid (BCA) protein detection kit. Protein samples (20 *μ*g/well) were separated on a 10% sodium dodecyl sulfate-polyacrylamide gel electrophoresis (SDS-PAGE) gel, transferred to a polyvinylidene fluoride (PVDF) membrane, and thereafter blocked in 5% bovine serum at room temperature for 2 h. The membranes were incubated with the primary antibodies against SIRT1 (11kDa, 1 : 1000), HIF-1a (1 : 1000), Egr-1 (80kDa, 1 : 1000), TGF-*β*1 (1 : 1000), and GAPDH (1 : 5000) overnight at 4°C. After being washed with a Tris-buffered saline and Tween 20 (TBST) buffer three times, the membrane was incubated with horseradish peroxidase- (HRP-) labeled IgG (1 : 10000) at room temperature for 2 h. Following a three-time wash with TBST, the protein bands were detected using enhanced chemiluminescence (ECL) reagents to avoid light and color and take photos in gel electrophoresis apparatus. The gray value was measured by image processing software, and the relative expression level of the target protein was calculated by the gray value. GAPDH was used as an internal reference.

### 2.10. Immunohistochemistry

The kidney and liver tissue samples were fixed, embedded in paraffin, and dewaxed and rehydrated in graded alcohols. This was followed by quenching of endogenous peroxidases and antigen retrieval. Nonspecific protein binding was inhibited with the serum. The experiment was performed using a primary antibody followed by an appropriate secondary antibody. Phosphate-buffered saline (PBS) was used as the negative control, and brownish yellow particles were treated as positive signals.

### 2.11. Transmission Electron Microscopy

Kidney tissues were fixed with 2.5% glutaraldehyde for 2 h, rinsed overnight, and fixed with 1% osmic acid for 2 h. Thereafter, the tissues were dehydrated in gradient pyruvate acid and stained with lead and uranium. The ultrastructure was observed under a transmission electron microscope.

### 2.12. Hematoxylin/Eosin (HE) Staining

The kidney and liver tissues were fixed in 4% paraformaldehyde for 48 h, embedded in paraffin, and stained with hematoxylin/eosin (HE) for histopathological observation.

### 2.13. Statistical Analysis

Experimental data were analyzed using SPASS25.0 software. The quantitative data were expressed as mean ± standard deviation. Differences between the two groups were analyzed using independent *t*-tests. *P* values < 0.05 were considered to be statistically significant.

## 3. Results

### 3.1. Fasting Blood Glucose and 24-Hour Urine Microalbumin Changes

**Figure 1:**
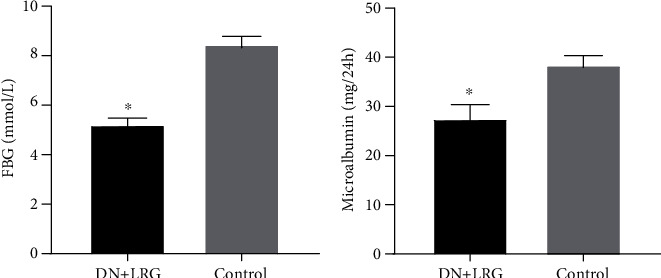
Fasting blood glucose and 24-hour microalbumin content. Control: diabetic nephropathy group; DN+LRG: diabetic nephropathy+liraglutide group; FBG: fasting blood glucose. ^∗^*P* < 0.05 vs. the control.

Following treatment with liraglutide, the blood glucose levels were significantly decreased in the DN rats (*P* < 0.05) (Figure 1(a)). Additionally, the 24-hour urine microalbumin content was also significantly reduced in the liraglutide-treated DN rats when compared to the control group (*P* < 0.05) (Figure 1(b)).

### 3.2. Changes in Biochemical Indices

**Figure 2:**
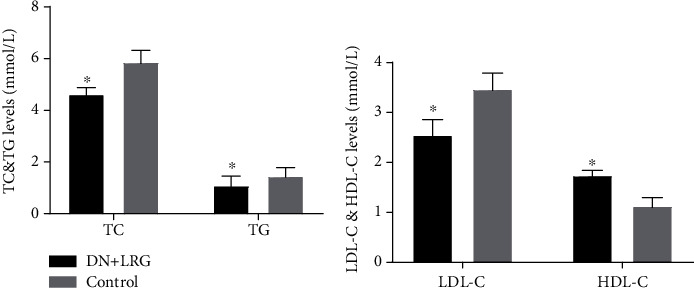
TC, TG, LDL-C, and HDL-C levels. Control: diabetic nephropathy group; DN+LRG: diabetic nephropathy+liraglutide group; TC: total cholesterol; TG: triglyceride; LDL-C: low-density lipoprotein; HDL-C: high-density lipoprotein. ^∗^*P* < 0.05 vs. the control.

**Figure 3:**
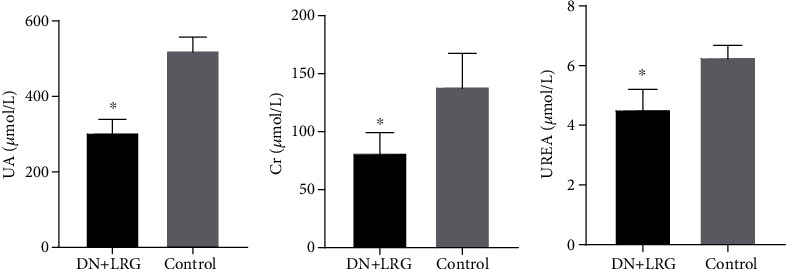
UA, Cr, and UREA levels. Control: diabetic nephropathy group; DN+LRG: diabetic nephropathy+liraglutide group; Cr: creatinine; UA: uric acid. ^∗^*P* < 0.05 vs. the control.

**Figure 4:**
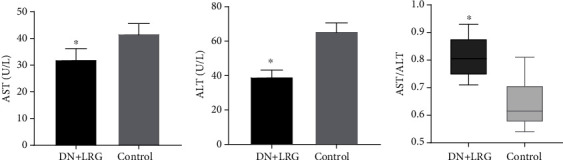
Levels of transferases in the blood. Control: diabetic nephropathy group; DN+LRG: diabetic nephropathy+liraglutide group; ALT: alanine aminotransferase; AST: aspartate aminotransferase. ^∗^*P* < 0.05 vs. the control.

Treatment with liraglutide resulted in a significant decrease in the serum levels of TC, TG, and LDL-C (*P* < 0.05) (Figures 2(a) and 2(b)); conversely, the level of HDL-C was increased (*P* < 0.05) in DN rats when compared with the control group (Figure 2(b)). Furthermore, the levels of blood UA, Cr, and UREA were significantly reduced in the liraglutide-treated DN rats than in the control group (*P* < 0.05) (Figure 3). AST and ALT levels were also significantly decreased (Figures 4(a) and 4(b)), and the AST/ALT ratio tended to be within the normal range (Figure 4(c)) following liraglutide treatment.

### 3.3. Liraglutide Inhibited the Expression of miR-34a in the Kidney of DN Rats

**Figure 5:**
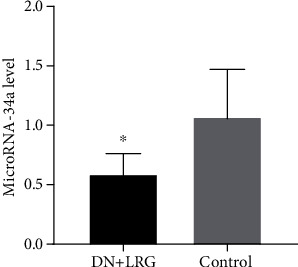
Expression of microRNA-34a in the kidneys. Control: diabetic nephropathy group; DN+LRG: diabetic nephropathy+liraglutide group. ^∗^*P* < 0.05 vs. the control.

Through quantitative real-time PCR analyzing the miR-34a of extracted tissues, we found that compared to the control DN group, the expression of miR-34a was significantly decreased in the kidney tissues of the DN rats treated with liraglutide (*P* < 0.05) (Figure 5).

### 3.4. Effect of Liraglutide on SIRT1 Downstream of miR-34a and Changes in Related Factors HIF-1a, Egr-1, and TGF-*β*1 in the Kidney of DN Rats

**Figure 6:**
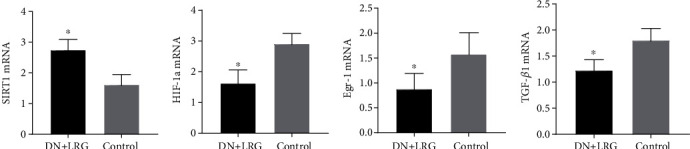
Expression levels of SIRT1, HIF-1a, Egr-1, and TGF-*β*1 mRNAs in the kidney. Control: diabetic nephropathy group; DN+LRG: diabetic nephropathy+liraglutide group; Egr-1: early growth response-1; HIF-1a: hypoxia-inducible factor-1 alpha; TGF-*β*1: transforming growth factor-*β*1. ^∗^*P* < 0.05 vs. the control.

**Figure 7:**
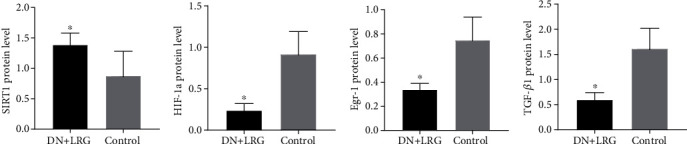
Expression of SIRT1, HIF-1a, Egr-1, and TGF-*β*1 proteins in the kidney. Control: diabetic nephropathy group; DN+LRG: diabetic nephropathy+liraglutide group; Egr-1: early growth response-1; HIF-1a: hypoxia-inducible factor-1 alpha; TGF-*β*1: transforming growth factor-*β*1. ^∗^*P* < 0.05 vs. the control.

**Figure 8:**
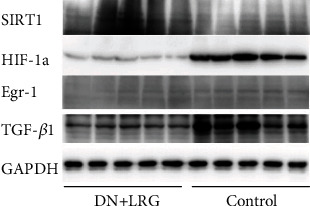
Western blot of SIRT1, HIF-1a, Egr-1, and TGF-*β*1 protein expression in the kidney. Control: diabetic nephropathy group; DN+LRG: diabetic nephropathy+liraglutide group; Egr-1: early growth response-1; HIF-1a: hypoxia-inducible factor-1 alpha; TGF-*β*1: transforming growth factor-*β*1.

Compared to the control group, the mRNA and protein levels of SIRT1 were significantly increased (Figures 6(a), 7(a), and 8), whereas the mRNA and protein levels of HIF-1a, Egr-1, and TGF-*β*1 were found to be decreased (*P* < 0.05) (Figures 6(b)–6(d), 7(b)–7(d), and 8) in the kidney tissues of liraglutide-treated DN rats.

**Figure 9:**
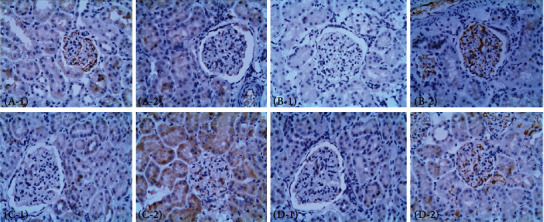
Immunohistochemical detection of SIRT, HIF-1a, Egr-1, and TGF-*β*1 in the kidney (×400). (a–d) SIRT1, hypoxia-inducible factor-1 *α* (HIF-1a), early growth response-1 (Egr-1), and transforming growth factor-*β*1 (TGF-*β*1), respectively; 1 and 2 represent the DN+LRG and control groups, respectively. Control: diabetic nephropathy group; DN+LRG: diabetic nephropathy+liraglutide group. ^∗^*P* < 0.05 vs. the control.

Immunohistochemical staining showed that the expression level of SIRT1 in the renal tubules and glomeruli was significantly increased following treatment with liraglutide (Figure 9(a)); conversely, the levels of HIF-1a, Egr-1, and TGF-*β*1 were decreased in the renal tubules and glomeruli of liraglutide-treated rats (Figures 9(b)–9(d)).

### 3.5. Ultrastructural and Pathological Changes in the Kidney of Liraglutide-Treated DN Rats

**Figure 10:**
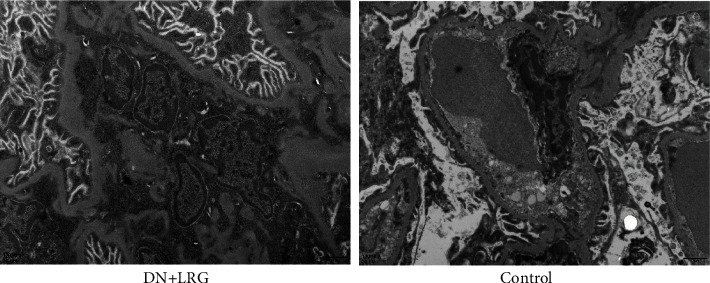
Changes of the renal structure detected by electron microscopy. Control: diabetic nephropathy group; DN+LRG: diabetic nephropathy+liraglutide group.

In the liraglutide-treated rats, we observed a slight widening of the mesangial area, an occasional appearance of deposits, mild edema of endothelial cells, and well-arranged foot processes in the kidney tissues (Figure 10). In the DN rats, electron microscopy revealed mesangial cell hyperplasia with lipid droplets, a widening of the mesangial area, low-density electron deposits, a shrunken basement membrane, a vascular cavity filled with a large number of red blood cells, and fusion foot processes (Figure 10).

**Figure 11:**
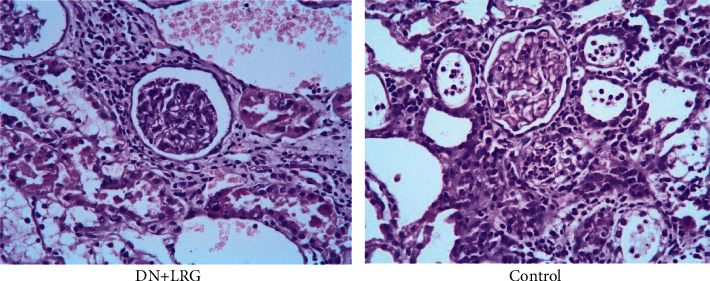
Hematoxylin eosin staining of the kidney (×400). Control: diabetic nephropathy group; DN+LRG: diabetic nephropathy+liraglutide group.

HE staining revealed that the kidney of liraglutide-treated rats adopts a more regular morphology of the glomerulus and Bowman sac cavity and lighter tubular edema (Figure 11). Conversely, the DN rats exhibited obvious pathological changes including irregular glomerular morphology, adhesion of the wall and visceral layer of the Bowman sac, an increased number of cells, and renal tubular edema with partial necrosis (Figure 11).

### 3.6. Liraglutide Inhibited the Expression of miR-34a in the Liver of DN Rats

**Figure 12:**
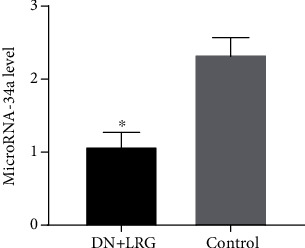
MicroRNA-34a expression in the livers. Control: diabetic nephropathy group; DN+LRG: diabetic nephropathy+liraglutide group. ^∗^*P* < 0.05 vs. the control.

Through quantitative real-time PCR analyzing the miR-34a of extracted tissues, we found that compared to the control group, liraglutide treatment significantly decreased the expression of miR-34a in the liver tissues (*P* < 0.05) (Figure 12).

### 3.7. Effect of Liraglutide on SIRT1 Downstream of miR-34a and Changes in Related Factors HIF-1a, Egr-1, and TGF-*β*1 in the Liver of DN Rats

**Figure 13:**
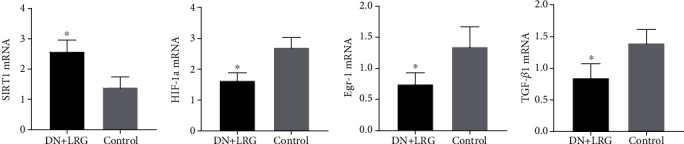
Expression of SIRT1, HIF-1a, Egr-1, and TGF-*β*1 mRNAs in the liver. Control: diabetic nephropathy group; DN+LRG: diabetic nephropathy+liraglutide group; Egr-1: early growth response-1; HIF-1a: hypoxia-inducible factor-1 alpha; TGF-*β*1: transforming growth factor-*β*1. ^∗^*P* < 0.05 vs. the control.

**Figure 14:**
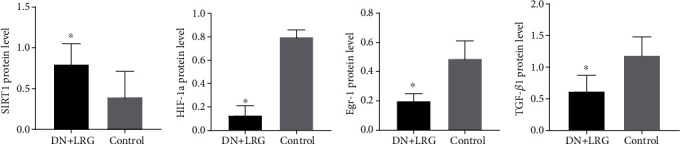
Expression of SIRT1, HIF-1a, Egr-1, and TGF-*β*1 proteins in the liver. Control: diabetic nephropathy group; DN+LRG: diabetic nephropathy+liraglutide group; Egr-1: early growth response-1; HIF-1a: hypoxia-inducible factor-1 alpha; TGF-*β*1: transforming growth factor-*β*1. ^∗^*P* < 0.05 vs. the control.

**Figure 15:**
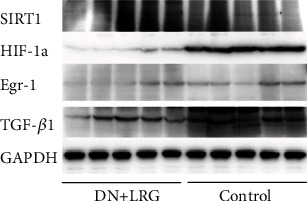
Western blot of SIRT1, HIF-1a, Egr-1, and TGF-*β*1 protein expression in the liver. Control: diabetic nephropathy group; DN+LRG: diabetic nephropathy+liraglutide group; Egr-1: early growth response-1; HIF-1a: hypoxia-inducible factor-1 alpha; TGF-*β*1: transforming growth factor-*β*1.

mRNA and protein levels of SIRT1 were significantly enhanced in liraglutide-treated rats compared to the control DN group (Figures 13(a), 14(a), and 15). In contrast, the expression levels of HIF-1a, Egr-1, and TGF-*β*1 mRNAs in the liver tissues were decreased (*P* < 0.05) (Figures 13(b)–13(d), 14(b)–14(d), and 15).

**Figure 16:**
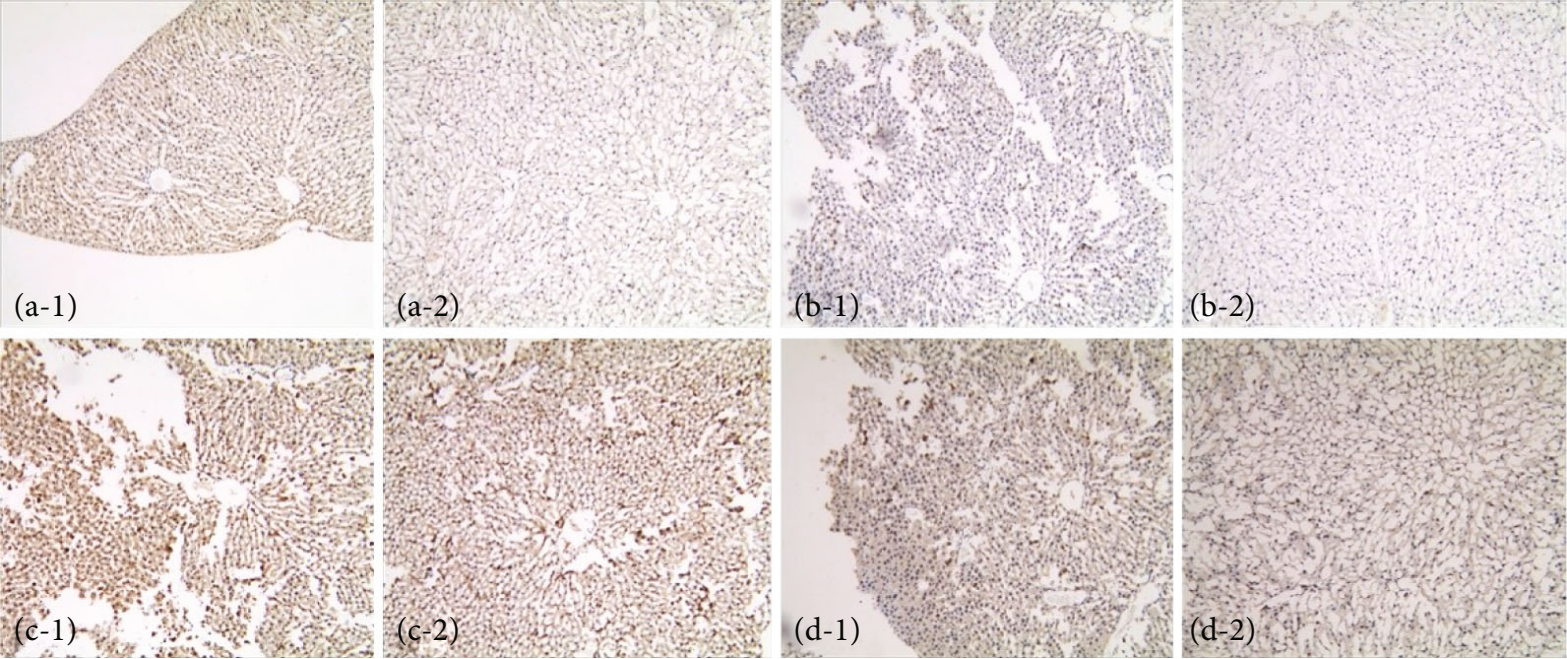
Immunohistochemical detection of SIRT1, HIF-1a, Egr-1, and TGF-*β*1 in the liver (×100). (a–d) SIRT1, HIF-1a, Egr-1, and TGF-*β*1, respectively. 1 and 2 indicate the DN+LRG and control groups, respectively. Control: diabetic nephropathy group; DN+LRG: diabetic nephropathy+liraglutide group.

Immunohistochemical staining showed that the expression level of SIRT1 was significantly increased in hepatocytes following the treatment of DN rats with liraglutide (Figure 16(a)); conversely, decreased levels of HIF-1a, Egr-1, and TGF-*β*1 in hepatocytes were detected in liraglutide-treated rats when compared to the control group (Figures 16(b)–16(d)).

### 3.8. Pathological Changes in the Liver of Liraglutide-Treated DN Rats

**Figure 17:**
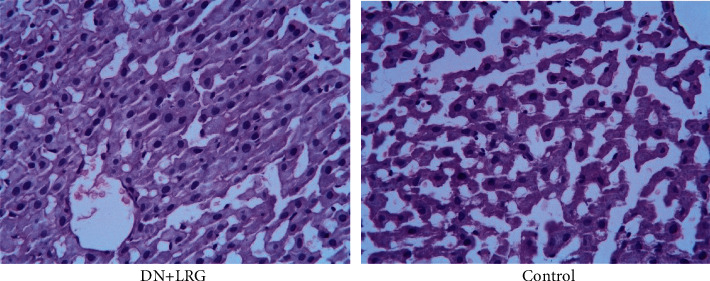
Hematoxylin eosin staining of the liver (×400). Control: diabetic nephropathy group; DN+LRG: diabetic nephropathy+liraglutide group.

In the liraglutide-treated DN rats, liver sinusoids were partially dilated (Figure 17). Conversely, disordered hepatic sinusoids with obvious dilation were detected in the liver of the control group (Figure 17).

## 4. Discussion

This study sought to investigate if liraglutide has a protective effect on the kidney and liver of DN rats that is mediated by the miR-34a/SIRT1 pathway. To accomplish this, we established a DN rat model using STZ and explored the effects of liraglutide on the expression of miR-34a and SIRT1 in the kidney and liver tissues. As reported, GLP-1 is expressed by the glucagon gene; in islet *α* cells, glucagon is the main expression product of the glucagon gene; in intestinal mucosal L cells, prohormone converting enzyme (PC1) can cleave glucagon to its carboxyl-terminal peptide sequence, namely, GLP-1. GLP-1 can stimulate the proliferation and differentiation of islet *β* cells, inhibit the apoptosis of islet *β* cells, and inhibit the release of glucagon; GLP-1 also has glucose concentration-dependent hypoglycemic effect [30, 31]. Liraglutide, a GLP-1 analog, is reported to delay gastric emptying, reduce food intake, and maintain stable blood glucose levels by promoting a glucose-dependent secretion of insulin and inhibiting the release of glucagon. Meanwhile, liraglutide also alleviates blood lipid levels in obese patients with T2DM and controls weight gain [32, 33], and compared with exenatide, liraglutide has higher safety and tolerance and more significant effect in improving beta cell function and insulin sensitivity [34]. Moreover, liraglutide could decrease the incidence of proteinuria in diabetic patients, reduce urine protein through anti-inflammatory and antioxidative stress activities, improve kidney function, and prevent the occurrence of DN and acute kidney injury [35]. Multiple studies have shown that miR-34a is closely associated with the occurrence of diabetes; miR-34a is significantly increased in the peripheral blood mononuclear cells in patients with T2DM and is correlated positively with LDL-C/HDL-C and negatively with TG/HDL-C [36]. TGF-*β*1 stimulation promotes the resynthesis of miR-34a in fibroblasts and increases miR-34a expression in microvessels; hence, TGF-*β*1 has a role in renal tubule-interstitial fibrosis [37]. Exogenous injection of miR-34a-containing microvesicles in mice promotes apoptosis of renal tubular cells, which in turn is closely related to renal fibrosis. In the presence of high glucose, downregulation of miR-34a inhibits the proliferation of mesangial cells; miR-34a regulates early mesangial proliferation and glomerular hypertrophy in DN by targeting the growth arrest-specific protein 1 (GAS1) [38]. Silent information regulator 1 (SIRT1), a nicotinamide adenine dinucleotide- (NAD^+^-) dependent deacetylase, plays an important role in the pathophysiology of kidney disease [39]. High glucose could induce HIF-1a expression, leading to the apoptosis of renal tubular epithelial cells; the polymorphism of HIF-1a Pro582Ser has differential effects on DN [40, 41]. High levels of Egr-1 result in an increase in the expression of profibrotic protein and influence DN development [42]. In glomerular mesangial cells cultured with high glucose, activation of the miR-34a/SIRT1 pathway upregulates the expression of HIF-1a and Egr-1, resulting in the overexpression of profibrotic factors, such as TGF-*β*1 and type IV collagen, in glomerular mesangial cells [23, 24].

In the present study, we detected changes in the biochemical indices and levels of miR-34a, SIRT1, HIF-1a, Egr-1, and TGF-*β* and explored whether liraglutide plays a protective role in DN rats through the regulation of the miR-34a/SIRT1 pathway. Our findings indicate a significant decrease in the levels of blood glucose, 24-hour urine microalbumin, TC, TG, LDL-C, UA, Cr, and UREA, while the HDL-C content was observed to be increased following liraglutide treatment. The data showed miR-34a expression to be significantly lower while SIRT1 levels were shown to be higher in the kidney of liraglutide-treated rats than control rats (*P* < 0.05); the mRNA and protein levels of HIF-1a, Egr-1, and TGF-*β*1 were significantly decreased following liraglutide treatment (*P* < 0.05). Immunohistochemical staining showed an obvious increase in SIRT1 levels while the levels of HIF-1a, Egr-1, and TGF-*β*1 were decreased in the renal tubules and glomeruli of DN rats (*P* < 0.05). Electron microscopy detected a slight widening of the mesangial area, occasional deposits, mild edema of endothelial cells, and well-arranged foot processes in the kidney tissues. In the DN rats, electron microscopy revealed mesangial cell hyperplasia with lipid droplets, a widening of the mesangial area, low-density electron deposits, a shrunken basement membrane, a large number of red blood cells filling the vascular cavity, and fusion foot processes. HE staining revealed that the kidney in liraglutide-treated rats had a more regular morphology of the glomerulus and Bowman sac cavity and lighter tubular edema. Conversely, the DN rats exhibited obvious pathological changes including irregular glomerular morphology, adhesion of the Bowman sac wall and visceral layer, increased number of cells, and renal tubular edema with partial necrosis. These findings suggest that liraglutide plays a protective role in the kidney of DN rats by decreasing the blood glucose/lipid levels and expression of miR-34, HIF-1a, Egr-1, and TGF-*β*1 while increasing the SIRT1 levels. Additionally, liraglutide treatment alleviates oxidative stress-induced injury in ZDF-induced DN rats [14]. During the process of epithelial-mesenchymal transition (EMT), inhibition of the miR-34a/SIRT1 pathway downregulates TGF-*β*1 expression in hepatocytes, thus playing an antifibrotic role [18]. HIF-1a is not only involved in kidney injury in DN but also closely related to inflammatory reactions and glucagon sensitivity in the liver [43]. Similarly, Egr-1 is not only involved in high glucose-induced mesangial cell injury but also associated with liver injury, which is mediated by various pathways [44, 45]. Therefore, our study further explored if liraglutide had a protective role in the liver by targeting the miR-34a/SIRT1 pathway. Our results showed that liraglutide treatment significantly decreased AST and ALT levels; moreover, the AST/ALT ratio also tended to be within the normal range. Data showed a significant decrease in the miR-34a and SIRT1 levels and suppressed HIF-1a, Egr-1, and TGF-*β*1 in the liver in DN rats (*P* < 0.05). Immunohistochemical staining showed that liraglutide treatment resulted in a significant increase in SIRT1 and a decrease in the protein levels of HIF-1a, Egr-1, and TGF-*β*1 in the hepatocytes of DN rats. From the staining of the liver with HE, a partial dilation of the liver sinusoids was observed in the liraglutide-treated DN rats. Conversely, a disordered hepatic sinusoid, which presented with obvious dilation, was detected in the control group, which had not received the liraglutide treatment. These findings suggest that liraglutide plays a protective role in the liver of DN rats by decreasing AST and ALT levels and miR-34a expression while increasing SIRT1 levels and inhibiting HIF-1a, Egr-1, and TGF-*β*1. Notably, the precise mechanism is unclear and requires further study.

## 5. Conclusion

In short, liraglutide plays a protective role in the kidney and liver of DN rats by decreasing miR-34a expression, increasing SIRT1 levels, and inhibiting HIF-1a, Egr-1, and TGF-*β*1. These findings provide a theoretical basis for the use of liraglutide in the prevention and treatment of DN.

## Figures and Tables

**Table 1:** Gene sequences of primers.

Gene	Forward primer	Reverse primer
SIRT1	5′-TGAAAGTAAGACCAGTAGC-3′	5′-AATGTAGATGAGGCAGAG-3′
HIF-1a	5′-CTTCACCCAGCAAGCGA-3′	5′-TGCAAGGCACAATATAGTCC-3′
Egr-1	5′-CTGGAGGAGATGATGC-3′	5′-GTAGTTTGGCTGGGAT-3′
TGF-*β*1	5′-GGCACCATCCATGACATGAACCG-3′	5′-GCCGTACACAGCACTTCTTCTCTG-3′
GAPDH	5′-CAAGTTCAACGGCACAG-3′	5′-CCAGTAGACTCCACGACAT-3′
miR-34a	5′-GGGTGGCAGTGTCTTAG-3′	5′-AACTGGTGTCGTGGAGTCGGC-3′
U6	5′-CTCGCTTCGGCAGCACATATACT-3′	5′-ACGCTTCACGAATTTGCGTGTC-3′

Note: Egr-1: early growth response-1; GADPH: glyceraldehyde 3-phosphate dehydrogenase; HIF-1a: hypoxia-inducible factor-1 alpha; TGF-*β*1: transforming growth factor-*β*1.

## Data Availability

The data analyzed during the study are not publicly available. Rigorous analysis of the data in order to ensure the objective authenticity of the results.
